# Isoxazolidine derivatives as corrosion inhibitors for low carbon steel in HCl solution: experimental, theoretical and effect of KI studies

**DOI:** 10.1039/c7ra11549k

**Published:** 2018-01-08

**Authors:** Mouheddin T. Alhaffar, Saviour A. Umoren, Ime. B. Obot, Shaikh A. Ali

**Affiliations:** Department of Chemistry, Faculty of Science, King Fahd University of Petroleum and Minerals Dhahran 31261 Saudi Arabia; Centre for Research Excellence in Corrosion, Research Institute, King Fahd University of Petroleum and Minerals Dhahran 31261 Saudi Arabia umoren@kfupm.edu.sa

## Abstract

Two isoxazolidine derivatives namely 5-(benzo[*d*][1,3]dioxol-5-ylmethyl)-2-tetradecyl isoxazolidine (BDMTI) and 5-(4-hydroxy-3-methoxybenzyl)-2-tetradecyl isoxazolidine (HMBTI) were synthesized and characterized using FTIR, C-NMR, H-NMR, and elemental analysis. The synthesized compounds were evaluated as corrosion inhibitors for API 5L X60 steel in 1 M HCl in the temperature range of 25–60 °C using gravimetric and electrochemical (Electrochemical Impedance Spectroscopy (EIS), Potentiodynamic Polarization (PDP) and Linear Polarization Resistance (LPR)) techniques. The effect of addition of a small amount of iodide ions on the corrosion inhibition performance of the compounds was also assessed. In addition, quantum chemical calculations and Monte Carlo simulations were employed to correlate the electronic properties of the compounds with the corrosion inhibition effect as well as to evaluate the adsorption/binding of the inhibitor molecules on the steel surface. Experimental results show that the two compounds inhibited the corrosion of carbon steel in an acid environment with HMBTI showing superior performance. The corrosion inhibition effect was found to be dependent on the inhibitors' concentration and temperature. Addition of iodide ions improves the inhibition efficiency considerably due to co-adsorption of the iodide ions and the inhibitors on the steel surface which was competitive in nature as confirmed from the synergistic parameter (*S*_1_) which was less than unity at higher temperature. Experimental and theoretical results are in good agreement.

## Abstract 2

Two isoxazolidine derivatives (BDMTI and HMBTI) were synthesized and characterized using FTIR, C-NMR, H-NMR and elemental analysis and evaluated as corrosion inhibitors for X60 steel in 1 M HCl solution.

## Graphical abstract

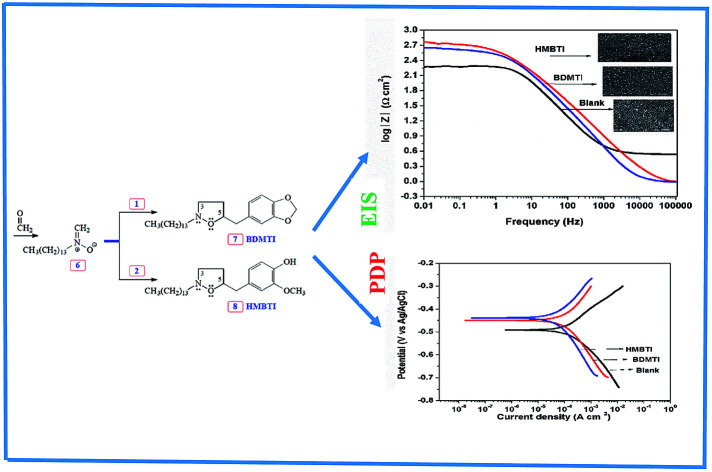

## 1 Introduction

The effective strategy adopted by the oil, gas and chemical industries in order to maximize profit and reduce cost is to use low carbon steel in place of expensive corrosion resistant alloys (CRAs) in their operations. However, the major problem associated with low carbon steel is its susceptibility to corrosion when it comes in contact with corrosive environments such as acids (HCl, H_2_SO_4_, and H_3_PO_4_), chloride rich solutions and aqueous hydrogen sulfide medium. The consequences of corrosion are enormous ranging from economic, health and safety to environmental standpoints amongst others.

Corrosion mitigation strategies adopted by some industries include materials selection, coatings and linings, cathodic protection and the use of corrosion inhibitors amongst others. The use of corrosion inhibitors is the most practical and cost effective method in fighting corrosion. Corrosion inhibitors retard corrosion by adsorbing onto the low carbon steel surface and blocking one or more of the electrochemical reactions occurring at the solution/metal interface. Well known corrosion inhibitors are organic compounds, including imidazolines, amides, amines and their derived salts.^1–3^ These organic compounds typically contain nitrogen, sulfur and oxygen, and hydrophobic hydrocarbon chains in their structures. However, inhibitors are usually effective only for a particular material in a certain environment,^4^ but the corrosion environments are highly variable; therefore, an inhibitor that works in one well may not work in another.^5^ Thus, it is necessary to continuously develop new formulas for different environment^6^ and numerous compounds are designed.^7,8^ Also, organic compounds alone are usually not effective enough and a proper mixture containing additional intensifiers, surfactants and solvents is needed.^9^

The functional groups in the organic molecules impart inhibition of corrosion.^10^ Isoxazolidines, known for many decades,^11–13^ are widely used in the synthesis of various natural products of biological interest.^14^ Relatively recently, the isoxazolidine functionality has been introduced to the corrosion literature for the first time.^15,16^ In the present work, we have synthesized two new isoxazolidines using cycloaddition reaction of nitrones and naturally occurring alkenes: eugenol and safrole, and studied their effects on the corrosion inhibition of low carbon steel in HCl solution using gravimetric and electrochemical techniques complemented with surface morphology characterization of the corroded steel samples without and with the synthesized inhibitors using SEM. Also, the influence of addition of a small amount of iodide ions on the corrosion inhibition performance of the isoxazolidines derivatives has also been assessed. The corrosion inhibition efficacy of the two new isoxazolidines derivatives BDMTI and HMBTI are correlated with quantum chemical parameters and Monte Carlo simulations with a view of understanding how the electronic properties of the compounds can influence the corrosion inhibition effect as well as the evaluating the adsorption/binding of the inhibitor molecules on the steel surface. The information derived from the computational studies will be highly useful in the design and synthesis of better performing corrosion inhibitors from this class of organic compound.

## 2 Experimental

### 2.1 Synthesis and characterization of isoxazolidines derivatives

#### 2.1.1 Materials

1-Bromotetradecane, hydroxylamine hydrochloride, triethylamine, silica gel 100, and paraformaldehyde (Fluka Chemie AG) and 1-tetradecene were used as received. Safrole (1) and eugenol (2) were purchased from Aldrich Co.

#### 2.1.2 Physical methods

A Perkin Elmer Series II Model 2400 and a Perkin Elmer (16F PC) spectrometer were used for elemental analyses and recording FTIR spectra, respectively. The NMR spectra were taken in CDCl_3_ on a 500 MHz JEOL LA spectrometer using TMS as standard.

### 2.2 Synthesis

#### 2.2.1 *N*-tetradecylhdroxylamine 4

To a mixture of hydroxylamine hydrochloride (35 g, 0.5 mol) in ethanol (250 cm^3^) was added triethylamine (46 g, 0.45 mol). After stirring using a magnetic stir bar at 20 °C for 10 min, 1-bromotetradecane 3 (20 g, 0.072 mol) was added in one portion and the resulting mixture was heated in a closed vessel for 7 h at 90 °C. The mixture was dumped onto water (600 cm^3^) and the white solid was filtered and washed with liberal excess of water. The white solid was taken in hot (50 °C) methanol and filtered, and the solid was washed with excess hot methanol. The white solid was crystallized from pet-ether to give the ditetradecylhydroxylamine 5 (3.5 g, 22.8%). The filtrate (∼700 cm^3^) was concentrated and the residual solid was crystallized from petroleum ether to give the white crystal of the tetradecylhydroxylamine 4 (9.5 g, 57.5%). Mp 94–95 °C; (found: C, 73.1; H, 13.4; N, 6.0. C_14_H_31_NO requires C, 73.30; H, 13.62; N, 6.11%); *ν*_max._ (KBr) 3263, 3158, 2918, 2851, 1654, 1512, 1466, 1379, 1151, 1062, 998, 970, 891, and 721 cm^−1^; *δ*_H_ (CDCl_3_) 0.88 (3H, t, *J* 7.0 Hz), 1.28 (22H, m), 1.52 (2H, quint, *J* 7.0 Hz), 2.93 (2H, t, *J* 7.0 Hz), NHOH protons are not observed presumably as a result of very broad signal; *δ*_C_ (CDCl_3_) 14.09, 22.70, 27.12, 27.21, 29.38, 29.60, 29.65, 29.69 (5C), 31.97, 54.12.

#### 2.2.2 5-(Benzo[*d*][1,3]dioxol-5-ylmethyl)-2-tetradecylisoxazolidine (7)

A mixture of hydroxylamine 4 (1.50, 6.5 mmol), paraformaldehyde (0.36 g, 12 mmol), and safrol (alkene) 1 (1.95 g, 12 mmol) in toluene (6 mL) was stirred using a magnetic stir bar at 110 °C in an RB flask at 110 °C under N_2_ for 7 h. After removal of solvent, the residual mixture was chromatographed over silica gel using 5 : 1 hexane/ether as eluant to give adduct 7 as a white solid (2.2 g, 84%). Mp 50––51 °C; (found: C, 74.1; H, 10.1; N, 3.4. C_25_H_41_NO_3_ requires C, 74.40; H, 10.24; N, 3.47%); *ν*_max._ (KBr) 3027, 2918, 2846, 1863, 1608, 1496, 1451, 1367, 1250, 1186, 1110, 1041, 932, 861, 813, 763, 723 and 662 cm^−1^; *δ*_H_ (CDCl_3_, −30 °C): 0.88 (3H, t, *J* 6.1 Hz), 1.24 (22, m), 1.57 (2H, m), 1.90–3.30 (8H, m), 4.21 (0.5H, quint, *J* 7.0 Hz), 4.31 (0.5H, quint, *J* 7.0 Hz), 5.96 (2H, s), 6.70 (3H, m); *δ*_C_ (CDCl_3_, −30 °C): 14.3, 22.8, 27.3, 27.5, 28.1, 29.5, 29.6, 29.7, 31.7, 31.9, 33.2, 40.5 (0.5), 40.9 (0.5C), 54.7 (0.5C), 55.2 (0.5C), 57.9 (0.5C), 59.1 (0.5C), 78.1, 100.8, 108.1, 109.7, 122.1, 131.4 (0.5C), 132.0 (0.5C), 145.7 (0.5C), 145.8 (0.5C), 147.2 (CDCl_3_ middle carbon: 77.1). The NMR signals indicated the presence of nitrogen invertomers in a 1 : 1 ratio equilibrating slowly on a NMR time scale.

#### 2.2.3 5-(4-Hydroxy-3-methoxybenzyl)-2-tetradecylisoxazolidine (8)

A mixture of hydroxylamine 4 (1.50, 6.5 mmol), paraformaldehyde (0.36 g, 12 mmol), and eugenol (2) (1.97 g, 12 mmol) in toluene (6 mL) in an RB flask was stirred using a magnetic stir bar at 110 °C under N_2_ for 7 h. After removal of solvent, the residual mixture was chromatographed over silica gel using 5 : 1 hexane/ether as eluant to give adduct 8 as a white solid (2.2 g, 88%). Mp 61–62 °C (found: C, 73.8; H, 10.5; N, 3.34. C_25_H_43_NO_3_ requires C, 74.03; H, 10.69; N, 3.45%); *ν*_max._ (KBr) around 3500 (broad), 2842, 2584, 1596, 1525, 1467, 1428, 1391, 1373, 1288, 1233, 1153, 1127, 1095, 1033, 1001, 959, 948, 924, 850, 798, 723, 673, 641, 567, 518 cm^−1^; *δ*_H_ (CDCl_3_, −30 °C) 0.87 (3H, m), 1.23 (22H, m), 1.54–1.64 (2H, m), 2.00 (1H, m), 2.26–2.92 (6H, m), 3.27–3.32 (1H, m), 3.85 (3H, m), 4.26 (0.5H, m), 4.37 (0.5H, m), 6.67–6.83 (3H, m), 7.90 (1H, OH); *δ*_C_ (CDCl_3_, −30 °C) 14.3, 22.8, 27.3, 27.4, 27.8, 29.5, 29.6, 29.7, 31.9, 33.1, 40.1 (0.5), 41.5 (0.5C), 54.4 (0.5C), 54.9 (0.5C), 55.5, 57.8 (0.5C), 58.9 (0.5C), 78.4, 111.6, 114.4, 121.5, 129.0 (0.5C), 129.4 (0.5C), 144.1, 146.5 (CDCl_3_ middle carbon: 77.1). The NMR signals indicated the presence of nitrogen invertomers in a 1 : 1 ratio equilibrating slowly on a NMR time scale.

### 2.3 Corrosion inhibition studies

#### 2.3.1 Materials preparation

The metal substrate utilized in the investigation was API 5L X60 steel with chemical composition as earlier reported.^17^ Prior to corrosion studies, the metal substrate was cut into coupons of dimensions 3 cm × 3 cm × 1 cm for weight loss measurements and 1 cm × 1 cm × 1 cm for electrochemical measurements. The coupons were abraded using successive grades of silicon carbide paper (#120–#1000), sonicated in ethanol bath for 10 min to remove the residues emanating from the grinding process, degreased with ethanol, rinsed with acetone and dried in warm air. Coupons for electrochemical studies were completely insulated leaving one side of the steel surface (area = 1 cm^2^) exposed. The prepared metal substrates were stored in desiccator prior to use. The corrosive medium was 1 M HCl prepared by diluting 37% analytical grade HCl (Sigma Aldrich) with double distilled water. The synthesized compounds BDMTI (7) and HMBTI (8) were used as test corrosion inhibitors in the concentration range of 20–100 ppm. Potassium iodide (KI) (Sigma-Aldrich) was added to the test inhibitors to evaluate synergistic inhibition effect at a concentration of 5 mM.

#### 2.3.2 Gravimetric measurements

The gravimetric measurements were performed following the ASTM standard procedures.^18^ The API 5L X60 steel samples were weighed and immersed in glass vessels containing 1 M HCl solution in the absence and presence of different concentrations of the BDMTI and HMBTI maintained at 25 °C to 60 °C, in a thermostated water bath for 24 h. After 24 h, the corroded steel samples were withdrawn, immersed in 1 M HCl for 20 seconds to loosen the corrosion products, thoroughly washed with distilled water, rinsed in acetone, dried in a stream of warm air and reweighed to determine the weight loss. The experiments were carried out in triplicates and only the average value of the weight losses were reported. The weight loss data were utilized to compute the corrosion rate and inhibition efficiency using eqn (1) and (2) respectively.1
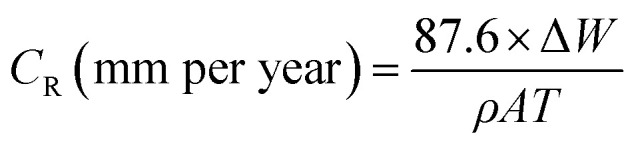
2
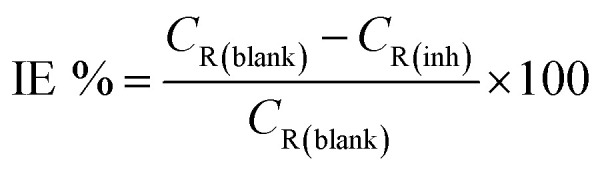
where *C*_R(blank)_ and *C*_R(inh)_ are the corrosion rates in the absence and presence of the inhibitor respectively, *W* is the average weight loss (mg), *ρ* is the density of the steel specimen (g cm^−3^), *A* is the surface area of the specimen (cm^2^) and *T* is the exposure time (h).

#### 2.3.3 Electrochemical measurements

Electrochemical measurements were undertaken in a three-electrode cell using Gamry Instrument Potentiostat/Galvanostat/ZRA (reference 600) with a Gamry framework system based on ESA410. Gamry applications include software DC105 for corrosion, EIS300 for electrochemical impedance spectroscopy (EIS) measurements and Echem Analyst 6.0 software package for data fitting. API 5L X60 samples, graphite rod and silver/silver chloride (Ag/AgCl) were used as working, counter and reference electrodes, respectively. All the measurements were taken after the working electrode was immersed for 1 h in the different test solutions at room temperature in order to attain a steady-state open-circuit potential (OCP). The frequency range from 100 kHz to 0.01 Hz with amplitude of 10 mV was used in electrochemical impedance experiments. The potentiodynamic polarization curves were recorded from cathodic potential of −150 mV to anodic potential of +100 mV at a scan rate of 0.5 mV s^−1^ with respect to free corrosion potential (*E*_corr_). The linear Tafel segments of the anodic and cathodic curves were extrapolated to corrosion potential to obtain the corrosion current densities (*i*_corr_) and other electrochemical parameters of interest. Linear polarization resistance (LPR) experiments were performed from −15 to +15 mV *versus E*_corr_ at the scan rate of 0.125 mV s^−1^.

Inhibition efficiency from electrochemical impedance spectroscopy (EIS), potentiodynamic polarization (PDP) and linear polarization resistance (LPR) was computed using eqn (3), (4) and (5) respectively.3
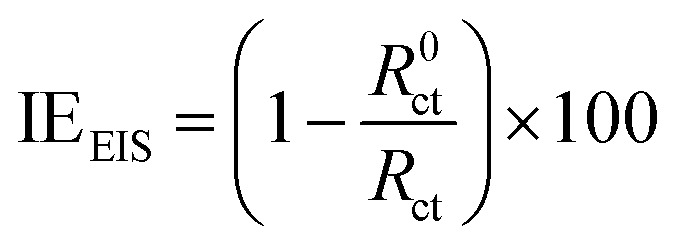
where *R*^0^_ct_ and *R*_ct_ are the charge transfer resistances in the absence and presence of the inhibitors respectively.4
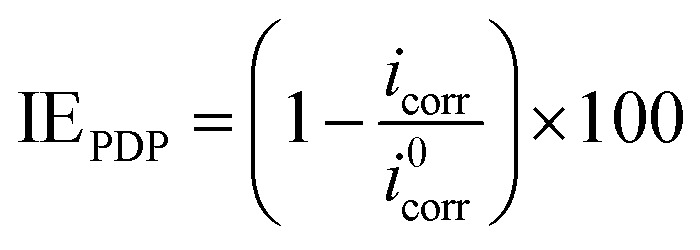
where *i*^0^_corr_ and *i*_corr_ are the corrosion current densities in the absence and presence of inhibitor respectively.5
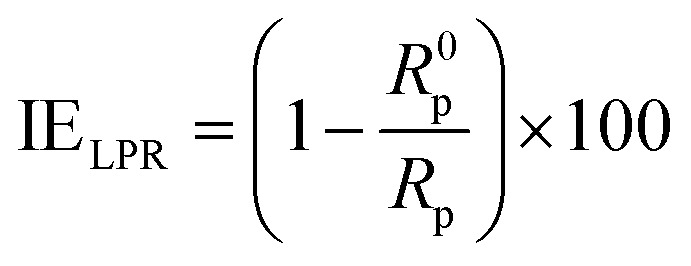
where *R*^0^_p_ and *R*_p_ are the polarization resistance values in the absence and presence of the inhibitor respectively.

### 2.4 Surface analysis

The surface analysis of the API 5L X60 steel was carried out using a JEOL JSM 6610- LV scanning electron microscope (SEM) operated at an acceleration voltage of 20 kV. Images of the specimens were recorded after 24 h exposure time in 1 M HCl without and with 60 ppm BDMTI, 60 ppm HMBTI and 60 ppm of each of the inhibitor in combination with 5 mM KI at 25 ± 1 °C.

### 2.5 Computational details

#### 2.5.1 Quantum chemical calculations

In this work, DFT based calculations were conducted by the DMol^3^ module implemented in the BOVIA Materials Studio (Version 8.0) from Accelyrs Inc. (San Diego, CA, USA). These calculations employed generalized gradient approximation functional (GGA) with double numerical basis set with polarization (DNP). The calculations were conducted in aqueous phase to simulate the effect of solvent. Highest occupied molecular orbitals (HOMO) and lowest unoccupied molecular orbitals (LUMO), which reveals the active sites of the molecules were plotted and discussed.

#### 2.5.2 Monte Carlo simulation

The strength of the interaction between BDMTI and HMBTI and Fe (110) surface was computed by using atomistic simulation as implemented in the adsorption locator module of the MS software. Metropolis Monte Carlo (MC) simulations methodology using the adsorption locator module by BIOVIA company. The MC calculation of the simulation of the interaction between the inhibitor molecules and Fe surface was carried out with a slab thickness of 5 Å, a supercell of (12 × 12) and vacuum of 50 Å with periodic boundary conditions to model a representative part of the interface devoid of any arbitrary boundary effects. For the whole simulation procedure, the COMPASS force field was used to optimize the structures for our systems. 100 water molecules (100H_2_O) were added to the simulation box to mimic the real corrosion environment.

## 3 Results and discussion

### 3.1 Synthesis of BDMTI and HMBTI

**Scheme 1:**
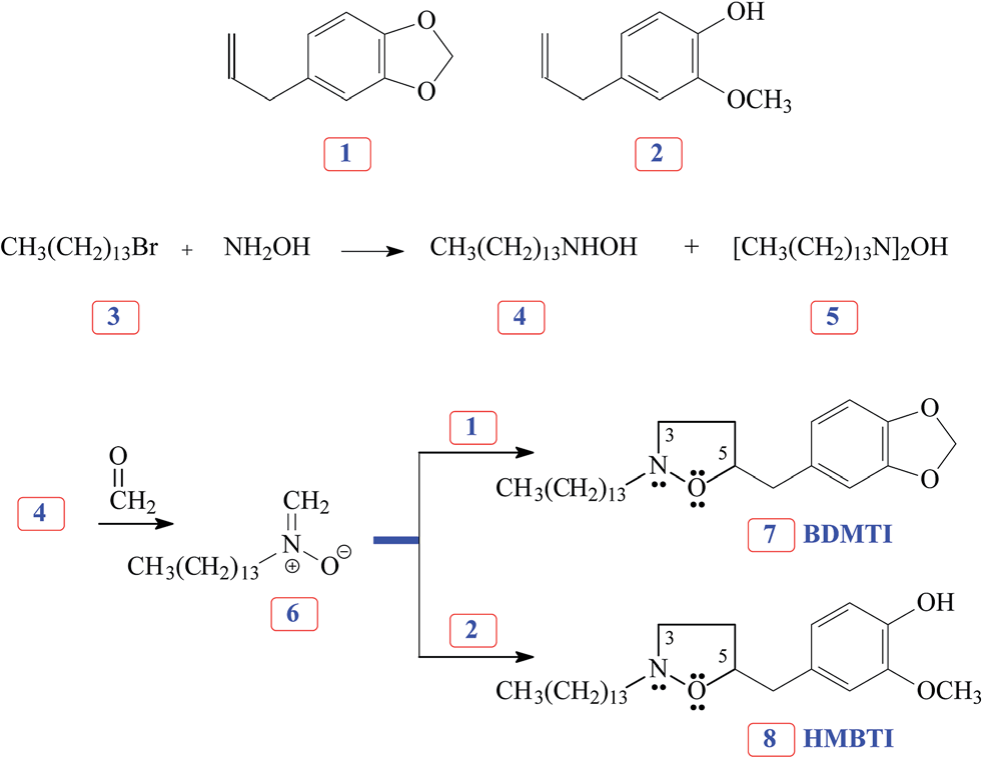
Synthesis of inhibitor molecules 7 and 8 using nitrone cycloaddition reaction.

The versatile nitrone cycloaddition reaction^11–13^ is the best chemical template for efficient synthesis of the isoxazolidines (Scheme 1). 1-Bromotetradecane 3 on treatment with excess hydroxylamine gave tetradecylhydroxylamine 4 as a major product. Nitrone 6, derived *via* condensation of hydroxylamine 4 with formaldehyde, underwent cycloaddition reaction with naturally occurring safrole 1 and eugenol 2 gave cycloadducts 7 and 8, respectively, in excellent yields. In each case, the cycloadduct was obtained regiospecifically; this regiochemical preference is well documented in the literature.^19^ The cycloadducts, characterized by spectral and elemental analyses, are obtained by attaching oxygen terminal of the nitrone to the more substituted internal carbon of the alkene.^20^ Note that the as-synthesized new inhibitor molecules 7 and 8 have isoxazolidine motifs, long hydrophobic tail as well as π-electron-rich aromatic groups which are potent corrosion inhibiting functionalities.

### 3.2 Gravimetric measurements

**Fig. 1:**
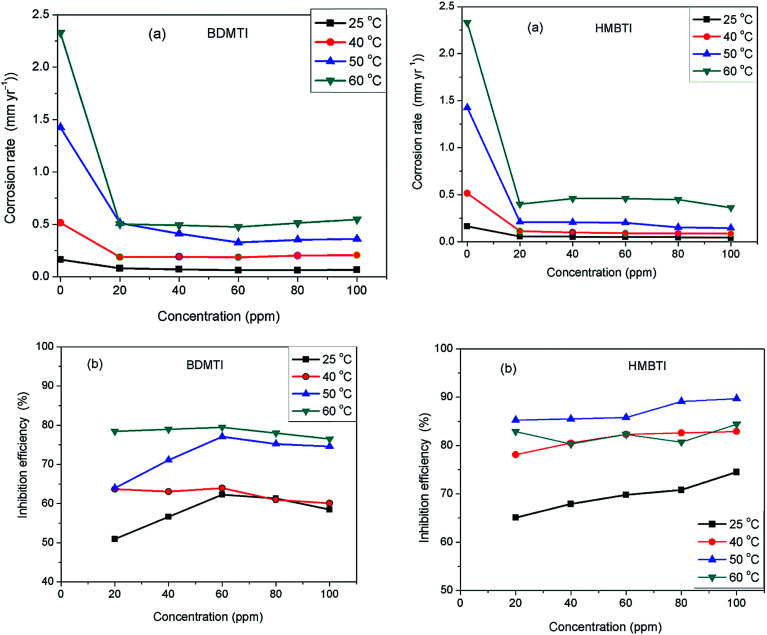
Variation of (a) corrosion rate and (b) inhibition efficiency with concentration for API 5L X60 steel without and with different concentrations of BDMTI and HMBTI in 1 M HCl solution at different temperatures from gravimetric measurements.

The technique of measuring the loss in mass or thickness of metal substrate with time when exposed to corrosive environment is still a reliable method in assessing corrosion of metals. Fig. 1(a) and (b) depict the corrosion rate and inhibition efficiency as a function of inhibitors (BDMTI and HMBTI) concentration respectively at the temperatures range of 25–60 °C. It is clear from the figures that corrosion rate decreased in the presence of the inhibitors compared to the blank solution. This is a clear indication that both BDMTI (7) and HMBTI (8) retard the dissolution of steel sample in the acid environment. It is also observed that corrosion rate is increased when the temperature is elevated from 25 to 60 °C both in the absence and presence of the inhibitors. This shows that the metal is susceptible to faster dissolution with increasing thermal agitation of the corrosive environment. With respect to variation of inhibition efficiency with concentration, close inspection of the figure reveals that for BDMTI, inhibition efficiency increased with increase in concentration up to 60 ppm, thereafter, further increase in concentration results in a decline in inhibition efficiency. Similar observation has been reported in the literature by some researchers.^21^ It was postulated that at the optimum inhibitor concentration, a maximum surface coverage was achieved and above this concentration, there may be a possibility of interaction between unadsorbed and adsorbed inhibitor molecules leading to desorption hence the decreased in IE with further increase in concentration. However, a different scenario is seen for HMBTI where inhibition efficiency increased with increase in concentration of the inhibitor. The increase in inhibition efficiency and the decrease in corrosion rates with the increase in concentration of inhibitor could be ascribed to the adsorption of the inhibitor molecules on the surface of steel and thus covered a certain area of the exposed electrode in HCl solution. By increasing the inhibitor concentration, the surface coverage was greatly increased due to the availability of more inhibitor molecules to be adsorbed on its surface. Temperature is also found to a play a significant role in the inhibitive performance of the two inhibitors. For BDMTI, it is noted that inhibition efficiency increases with increase in temperature while for HMBTI, inhibition efficiency increased with increase in temperature up to 50 °C and subsequently decline when the temperature is raised to 60 °C.

### 3.3 PDP and LPR measurements

**Fig. 2:**
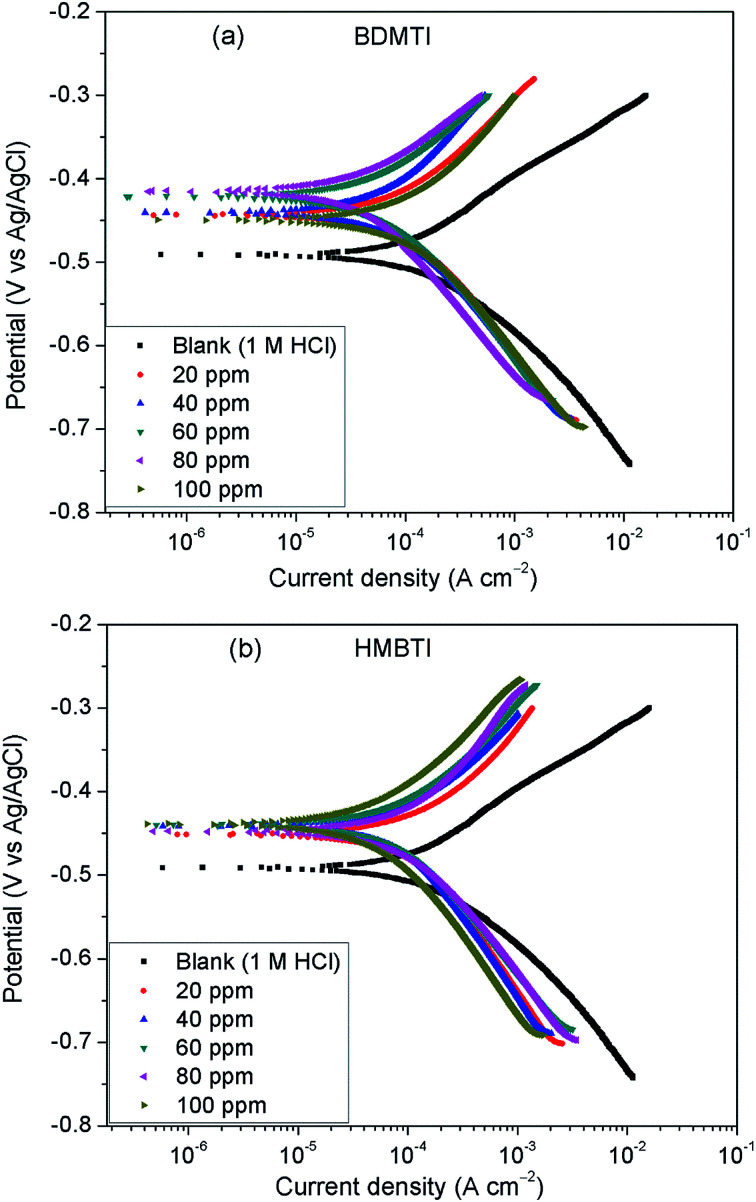
Potentiodynamic polarization plots for API 5L X60 steel without and with different concentrations of (a) BDMTI and (b) HMBTI at 25 °C.

To gain insights into the kinetics of anodic reaction (metal dissolution) and cathodic reaction (hydrogen evolution), PDP experiments were undertaken for API 5L X60 steel in 1 M HCl devoid of and in the presence of different concentrations of BDMTI and HMBTI at 25 °C. Fig. 2 depicts the polarization curves for (a) BDMTI and (b) HMBTI respectively. It can be deduced from the plots that the nature of the polarization curves are the same in both the uninhibited and inhibited solutions. However, addition of both BDMTI and HMBTI to the corrosive medium (1 M HCl) reduces the corrosion rate of the steel specimen as evidenced in the shifting of the corrosion current density to regions of lower values in comparison to the blank indicating corrosion inhibiting ability of the compounds. Examination of Fig. 2 also reveals that the corrosion potential (*E*_corr_) in the presence of both BDMTI and HMBTI shifted to noble values relative to the blank. It is also observed that the API 5L X60 undergo active dissolution within the range of potential investigated without inclination towards passivation both in the absence and presence of the inhibitors.

Electrochemical parameters obtained from the analysis of the polarization curves using extrapolation method are displayed in Table 1. It is clear from the table that corrosion current density decreased while the inhibition efficiency increased with increase in concentration of BDMTI up to 60 ppm. Beyond this concentration, an upward trend in corrosion current density and a downward trend in inhibition efficiency with a further increase in BDMTI concentration is observed. As regards HMBTI, a reduction in corrosion current density and an increase in inhibition efficiency is clearly visible from Table 1. This result is in agreement with that of gravimetric measurements. Again, it is noticed from Table 1 that there is notable changes in both the anodic and cathodic Tafel constants in the presence of inhibitors relative to the blank although with respect to different concentrations of the inhibitors, no definite trend is seen. This observation coupled with the fact that both anodic and cathodic current densities are reduced suggests that both BDMTI and HMBTI function as a mixed type corrosion inhibitors.^22^

**Table 1:** Potentiodynamic polarization (PDP) and linear polarization resistance (LPR) parameters for API 5L X60 steel in 1 M HCl without and with different concentrations of BDMTI and HMBTI at 25 °C

Inhibitor	Concentration (mg L^−1^)	*E*_corr_ (mV/Ag/AgCl)	*I*_corr_ (μA cm^−2^)	PDP method			LPR method	
				*β*_a_ (mV dec^−1^)	*β*_c_ (mV dec^−1^)	IE (%)	*R*_p_ (Ω cm^2^)	IE (%)
	Blank	−491	158.0	129.5	112.6	—	189.6	—
BDMTI	20	−444	102.0	146.1	153.4	35.4	310.9	39.0
	40	−440	90.7	193.9	153.8	42.6	448.2	57.7
	60	−421	52.0	114.2	131.2	67.1	497.9	61.9
	80	−445	111.0	163.5	181.2	30.4	361.3	47.5
	100	−445	127.0	151.9	236.7	19.6	291.4	34.9
HMBTI	20	−441	118	129.3	250.4	25.3	336.8	43.8
	40	−440	115	151.4	218.8	27.2	369.4	48.7
	60	−432	74.4	107.9	152.3	52.9	370.4	48.8
	80	−439	55.6	129.6	161.3	64.8	380.7	50.2
	100	−400	30.8	85.3	130.6	80.5	643.6	70.5

LPR method was also employed to assess the corrosion inhibition performance of BDMTI and HMBTI for API 5L X60 in 1 M HCl solution. The values of the polarization resistance (*R*_p_) and the computed inhibition efficiency obtained from LPR measurements are also listed in Table 1. From the table, *R*_p_ is observed to increase in inhibited solution compared to the blank indicating that BDMTI and HMBTI inhibited corrosion of steel in the acid medium. The trend of inhibition efficiency as a function of the inhibitors concentration noted for gravimetric and potentiodynamic polarization measurements is the same for LPR measurements. For BDMTI, the optimum inhibition efficiency (61.9%) was realized at 60 ppm and above this concentration, the IE declined to the lowest value of 34.9% at 100 ppm. For HMBTI, the IE increased with increasing concentration to reach an optimum value of 70.5% at 100 ppm concentration.

### 3.4 EIS measurements

**Fig. 3:**
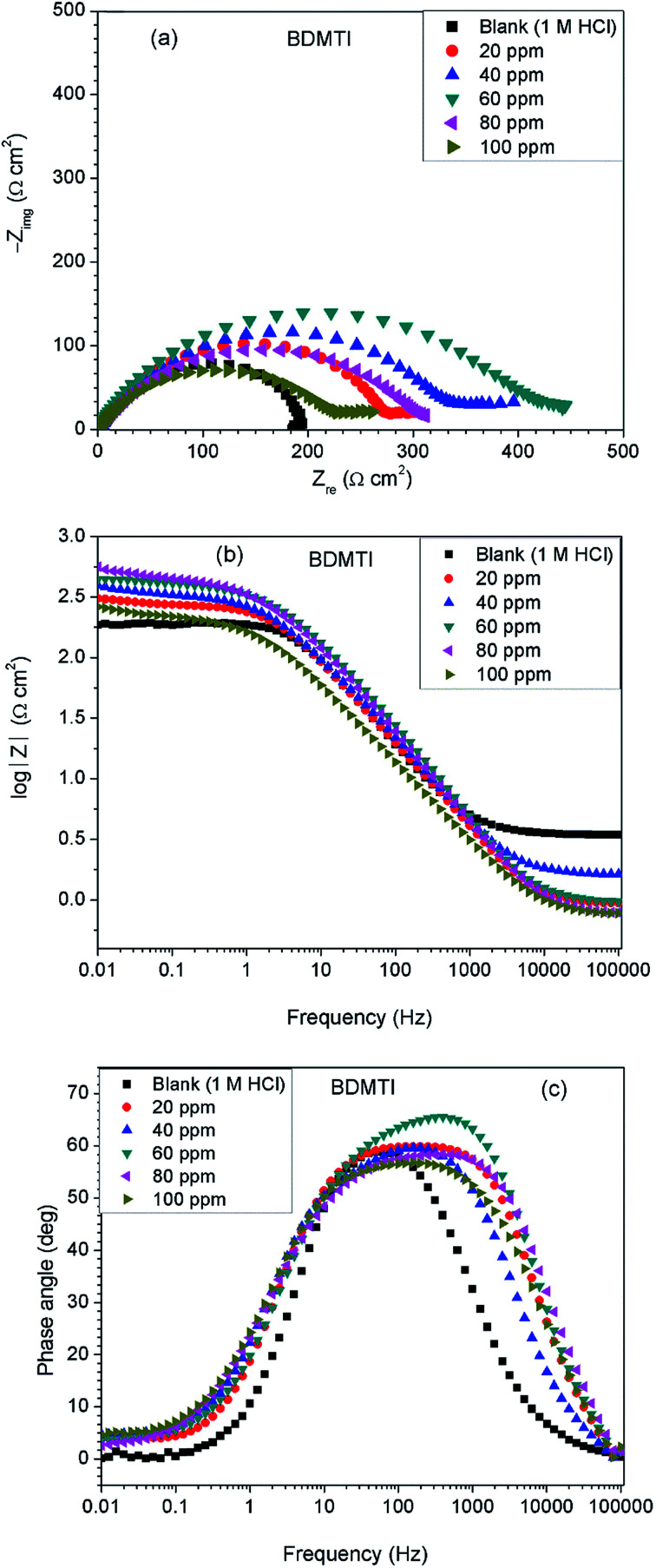
Impedance plots for API 5L X60 steel in 1 M HCl in the absence and presence of different concentrations of BDMTI exemplified as (a) Nyquist and (b) Bode modulus and (c) phase angle plots at 25 °C.

**Fig. 4:**
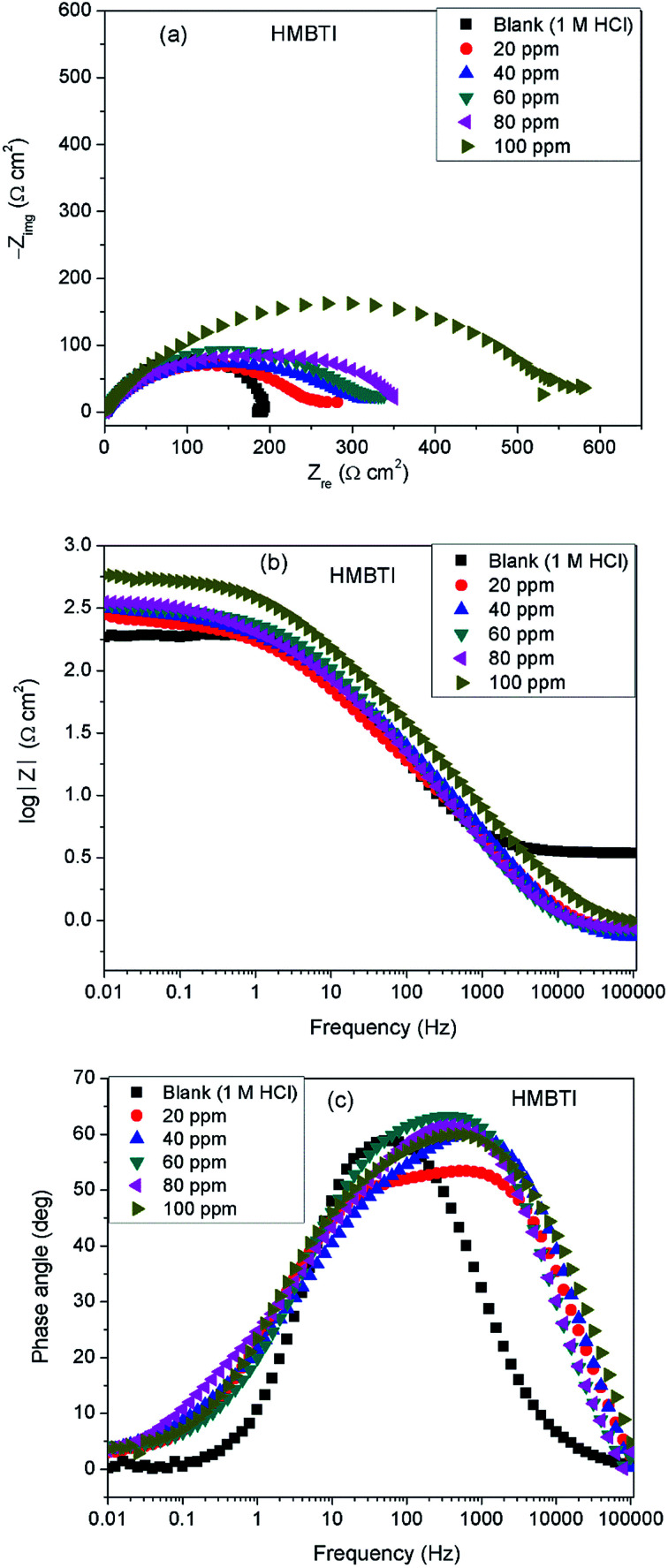
Impedance plots for API 5L X60 steel in 1 M HCl in the absence and presence of different concentrations of HMBTI exemplified as (a) Nyquist and (b) Bode modulus and (c) phase angle plots at 25 °C.

**Fig. 5:**
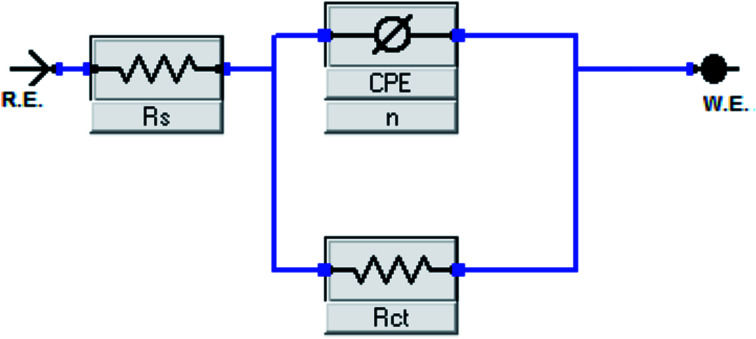
Equivalent circuit diagrams used to fit impedance data in the absence and presence of BDMTI and HMBTI.

Fig. 3 depicts impedance plots for API 5L X60 immersed for 1 h in 1 M HCl without and with different concentrations of BDMTI in (a) Nyquist, (b) Bode modulus and (c) phase angle representations. Similar plots for HMBTI are presented in Fig. 4. As can be clearly seen, the Nyquist plot in each case is composed of one depressed capacitive semicircle corresponding to one time constant in the Bode plot. The shape of the Nyquist plot is the same both in the absence and presence of the inhibitors indicating no change in the mechanism of the corrosion process. However, the diameter of the semicircle changes with addition of the inhibitors to the corrosive environment becoming larger as the concentration of the inhibitor increases. The two studied inhibitors displayed different kinds of behavior with respect to the growth of the Nyquist semicircles with increment in concentration. For BDMTI, the semicircle increased with increase in concentration up to 60 ppm and thereafter diminished with further increase in concentration; whereas HMBTI showed a progressive increase in the Nyquist semicircle with concentration up to 100 ppm. The depressed nature of the semicircle is a characteristic of solid electrode that show frequency dispersion which is often associated with diverse physical phenomena including surface roughness and inhomogeneities of solid electrode in the course of corrosion process.^23^ The Bode modulus displayed a single time constant which is consistent with the Nyquist spectra. The value of absolute Bode modulus is seen to shift to higher values in the low frequency region with increase in the concentration of the inhibitors, again pointing to effective corrosion inhibition by higher inhibitor concentrations than lower concentrations especially for HMBTI. The phase angles in all cases are moving towards 80° and thus eliminate possible diffusion control process.^24^ In modeling of corrosion systems, constant phase elements (CPE) is used in place of ideal capacitors to account for the non-homogeneity in the system.^25^ The impedance of a CPE is given by:

6 *Z*_CPE_ = *Y*_0_^−1^(j*ω*)^−*n*^

where *Y*_0_ is the CPE constant and *n* the CPE exponent; j = (−1)^1/2^ which is an imaginary number and *ω* is the angular frequency in rad s^−1^. The impedance data was analyzed using an equivalent circuit that models the physical phenomena taking place at the steel–solution interface. Fig. 5 depicts the equivalent circuit employed to simulate the experimental impedance data for the corrosion of API 5L X60 in 1 M HCl in the absence and presence of the inhibitors. The equivalent circuit consists of the solution resistance (*R*_s_), charge transfer resistance (*R*_ct_) and constant phase element (CPE). An excellent fit was obtained with this equivalent circuit as could been from the goodness of fit values (Table 2). Electrochemical impedance parameters generated from the fitting results are listed in Table 2. From Table 2, it is clearly seen that the charge transfer resistance exhibits an increasing trend with increasing BDMTI concentration up to 60 ppm and beyond this concentration, it decreases, while for HMBTI, it increases with increase in concentration within the range of concentration studied. The CPE exponent, *n* is a measure of surface inhomogeneity and it is observed to decrease in the presence of the inhibitors compared to the blank. Similar observation has been reported by Oguzie *et al.* and other workers^26,27^ which was attributed to an increase in heterogeneity resulting from the adsorption of the inhibitors on the metal steel surface.

**Table 2:** Electrochemical impedance spectroscopy parameters for API 5L X60 steel in 1 M HCl without and with different concentrations of BDMTI and HMBTI at 25 °C

Inhibitor	Concentration (mg L^−1^)	*R*_s_ (Ω cm^2^)	*R*_ct_ (Ω cm^2^)	*Y*_0_ (μΩ s^*n*^ cm^−2^)	*n*	*C*_dl_ (μF cm^−2^)	*χ* ^2^ × 10^−3^	IE (%)
	Blank	3.44	192.9	784.7	0.82	377.2	1.00	—
BDMTI	20	0.85	291.0	388.7	0.75	184.3	2.76	33.7
	40	1.51	353.3	276.4	0.74	147.6	2.68	45.4
	60	0.89	417.6	241.0	0.78	131.5	3.99	53.8
	80	0.69	307.7	403.0	0.72	208.9	3.46	37.3
	100	0.69	237.6	488.9	0.71	241.0	2.51	18.8
HMBTI	20	0.58	275.8	500.0	0.69	198.2	11.6	30.1
	40	0.76	303.1	343.3	0.76	177.3	5.74	36.4
	60	0.72	324.4	389.1	0.75	246.4	3.25	40.5
	80	0.81	547.9	299.8	0.70	150.6	3.15	64.8
	100	0.76	597.4	213.0	0.81	157.3	2.53	67.7

The values of double layer capacitance (*C*_dl_) were computed using the equation:^28^

7 *C*_dl_ = *Y*_0_(*ω*_max_)^*n*−1^

where *ω*_max_ = 2π*f*_max_, *f*_max_ is the frequency at which the imaginary component of the impedance is maximum. The *C*_dl_ values are observed to decrease in the presence of the inhibitors relative to the blank. In general, organic compounds have a much lower dielectric constant and a larger volume than water molecule.^29^ The decrease in *C*_dl_ values might be attributed to the gradual replacement of water molecules by the adsorption of the organic molecules at the steel/solution interface.^30^ The thickness of the protective film (*δ*) is related to *C*_dl_ according to the expression of the double layer capacitance presented in the Helmholtz model,^31^8
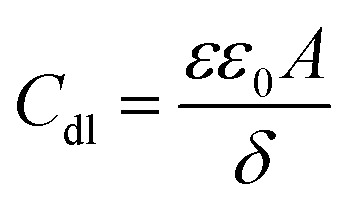
where *ε* is the dielectric constant of the medium, *ε*_0_ is the vacuum permittivity, *A* is the area of the electrode and *δ* is the thickness of the protecting layer.

### 3.5 Effect of iodide ions

**Fig. 6:**
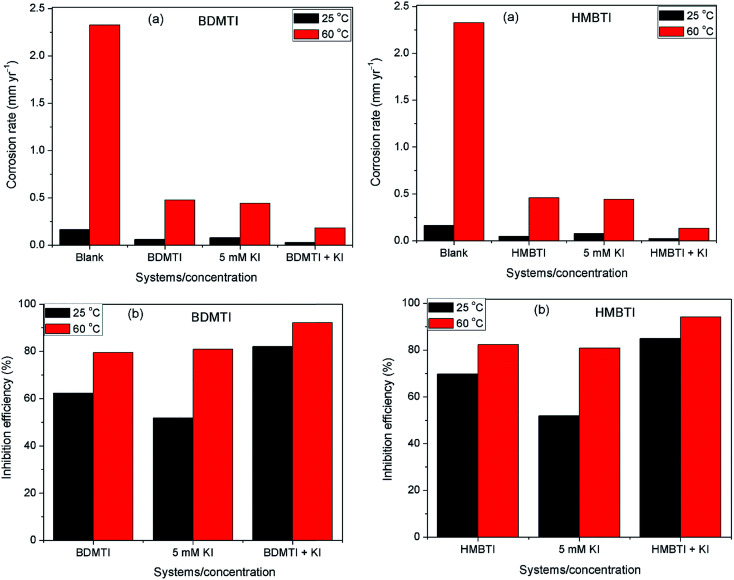
Plot showing (a) corrosion rate and (b) inhibition efficiency for API 5L X60 steel in 1 M HCl without and with KI, BDMTI, HMBTI, BDMTI + KI and HMBTI + KI at 25 and 60 °C from gravimetric measurements.

The corrosion rate and inhibition efficiency from weight loss measurements for API 5L X60 steel in 1 M HCl without and containing 60 ppm BDMTI, 60 ppm HMBTI, 5 mM KI, 60 ppm BDMTI + 5 mM KI and 60 ppm HMBTI + 5 mM KI at 25 and 60 °C are shown in Fig. 6(a) and (b) respectively. Inspection of the figure reveals that corrosion rate decreased when the two inhibitors and KI were added to the corrosive medium indicating that the corrosion of the steel was slowed down in the presence of the additives. Further reduction in corrosion rate is recorded in the presence of inhibitors – KI mixtures. Also, corrosion rate is observed to increase with increase in temperature from 25 to 60 °C in the absence and presence of the additives. The inhibition efficiencies in the presence of BDMTI, HMBTI and KI alone are 62.3, 69.8 and 51.9% respectively at 25 °C. The values obtained at 60 °C were 79.5, 82.3 and 80.9% respectively. In the presence of BDMTI + KI, the IE values were 82.1 and 92.2% at 25 and 60 °C respectively while for HMBTI, 84.9 and 94.2% inhibition efficiency were recorded at 25 and 60 °C respectively. These data clearly show that IE increases with increase in temperature. The increased inhibition efficiency of BDMTI and HMBTI in the presence of iodide ions shows the possible existence of synergism between the two inhibitors and iodide ions. However, in order to determine the existence of synergistic effect between BDMTI/HMBTI and iodide ions, the synergistic parameter (*S*_1_) given by the expression in eqn (9) was computed:^32–34^9
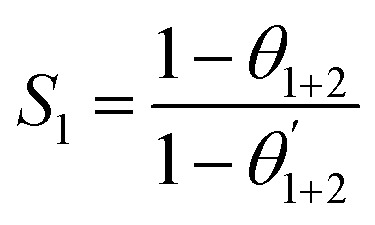
where *θ*_1+2_ = (*θ*_1_ + *θ*_2_) − (*θ*_1_*θ*_2_), *θ*_1_ is the surface coverage (*θ*) of BDMTI and HMBTI, *θ*_2_ is the surface coverage (*θ*) of KI and 
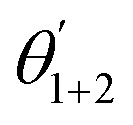
 is the combined surface coverage (*θ*) of BDMTI/HMBTI and KI. *S*_1_ greater than unity points to existence of synergism between the selected inhibitors combination whereas *S*_1_ less than unity indicates antagonistic effect which may lead to competitive adsorption and no interaction between the two inhibitors prevails when *S*_1_ is equal to unity. The synergistic parameter obtained for BDMTI were 1.01 and 0.50 at 25 and 60 °C respectively whereas the values obtained for HMBTI at the same temperatures were 0.96 and 0.5 respectively. From these results, it does appear that there was no interaction between the inhibitors and KI at 25 °C since the synergistic parameter is almost or close to unity in case of BDMTI and HMBTI respectively. At 60 °C, the synergistic parameter for both inhibitors is less than unity meaning that antagonistic rather than synergistic behavior between the inhibitors and KI prevails. The addition of iodide ions simply enhances the stability of both BDMTI and HMBTI adsorbed on the steel surface by co-adsorption mechanism suggested by some researchers.^26,35,36^ The co-adsorption effect which may be cooperative or competitive is competitive in this case where the anion (I^−^) and the inhibitors (BDMTI and HMBTI) are adsorbed on the different sites on the metal surface.

### 3.6 Adsorption isotherm

**Fig. 7:**
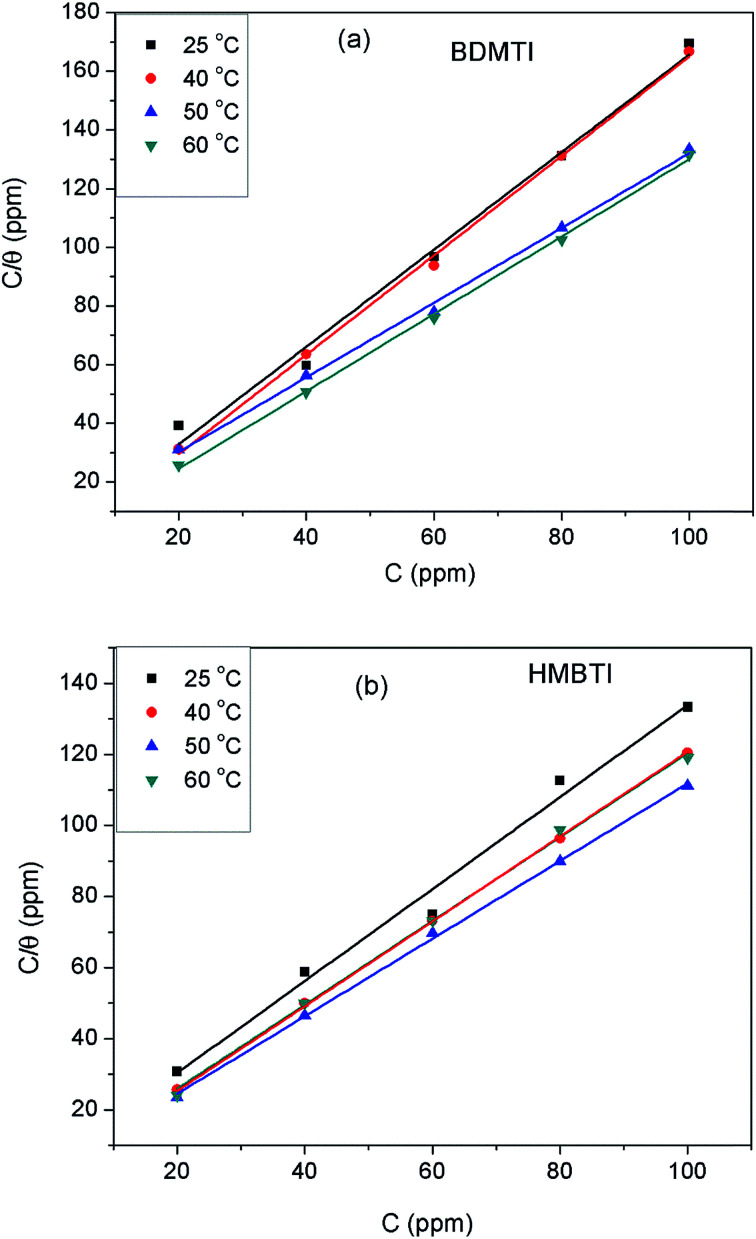
Langmuir adsorption isotherm for (a) BDMTI and (b) HMBTI on API 5L X60 steel in 1 M HCl at different temperatures.

The extent of corrosion inhibition is a function of the mode of adsorption of the inhibitor as well as the surface conditions.^37^ If it is assumed that the part of metal surface covered by inhibitor is immune to corrosion and the area not covered are prone to corrosion, that is, the blocking effect of the adsorbed species is solely responsible for corrosion inhibition effect; then the degree of surface coverage (*θ*) is indispensable in the construction of adsorption isotherm. In the present work, *θ* values were estimated from the expression: *θ* = IE/100 (assuming a direct relationship between surface coverage and inhibition efficiency) for different inhibitor concentrations from the weight loss measurement at different temperatures. The values obtained were theoretically fitted into different adsorption isotherm models and the correlation coefficient (*R*^2^) value was used to select the best fit isotherm. Langmuir isotherm was found to be the best fit adsorption isotherm model to describe the adsorption of both BDMTI and HMBTI onto the steel surface. Langmuir isotherm is characterized by the following expression:10
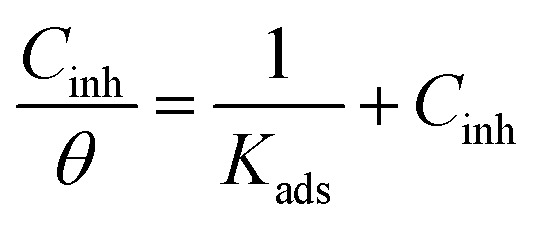
where *C*_inh_ is the inhibitor concentration and *K*_ads_ is the equilibrium constant of the adsorption–desorption process. Fig. 7 presents the plot of *C*_inh_/*θ* against *C*_inh_ using *θ* values obtained for gravimetric technique at various temperatures. In all cases, linear plots were obtained and the *R*^2^ values are close to one (Table 3) suggesting that the adsorption of BDMTI and HMBTI molecules onto the API 5L X60 steel surface obeys Langmuir isotherm. Although the plots are linear with good correlation coefficient values, the slopes are more than unity indicating a deviation from ideal Langmuir adsorption equation.^38^ This suggests that there is an interaction between adsorbed species of the inhibitor molecules on the metal surface. Langmuir equation had been derived on the assumption that no interaction exist among adsorbed inhibitor molecules. This is not true as many reports in the literature have shown that large molecules such as polymers^39,40^ and organic molecules having polar groups^41^ can interact by mutual repulsion or attraction.

**Table 3:** Langmuir adsorption parameters for API 5L X60 steel in 1 M HCl containing BDMTI and HMBTI from weight loss measurements at different temperatures

Inhibitor	Temperature (°C)	Δ*G*^0^_ads_ (kJ mol^−1^)	*K*_ads_ (L mg^−1^)	Slope	*R* ^2^
BDMTI	25	−37.01	3.07	1.66	0.988
	40	−32.13	0.23	1.69	0.998
	50	−32.91	0.21	1.27	0.997
	60	−36.49	0.53	1.32	0.999
HMBTI	25	−30.54	0.23	1.29	0.984
	40	−33.77	0.43	1.18	0.999
	50	−34.54	0.39	1.09	0.999
	60	−37.29	0.71	1.19	0.998

From the values of *K*_ads_, the standard free energy of adsorption for both BDMTI and HMBTI at different temperatures were obtained as follows:^32^

11 Δ*G*^0^_ads_ = −*RT* ln(1 × 10^6^*K*_ads_)

where *R* is the universal constant, *T* is the absolute temperature and 1 × 10^6^ is the concentration of water molecules expressed in mg L^−1^ or ppm. The computed values of Δ*G*^0^_ads_ listed in Table 3 are in the range −30.54 and −37.29 kJ mol^−1^. These values are less negative than −40 kJ mol^−1^ and more negative than −20 kJ mol^−1^. In the literature, the value of Δ*G*^0^_ads_ equal to −20 kJ mol^−1^ or less is taken to indicate physisorption involving electrostatic interaction between charged molecules whereas those in the order of −40 kJ mol^−1^ or more is interpreted as chemisorption involving charge sharing or transfer from the inhibitors to the metal surface to form a kind of co-ordinate bond.^32^ From the values of Δ*G*^0^_ads_ obtained in this present work, it can be deduced that adsorption mechanism of both BDMTI and HMBTI on API 5L X60 steel surface may involve two types of interactions namely physisorption and chemisorption. As pointed out by Lozano *et al.*^37^ as a consequence of strong adsorption of water molecules on the steel surface, it may be assumed that adsorption occurs first due to the physical forces. The replacement of the adsorbed water molecules is accompanied by a chemical interaction between the adsorbate (inhibitors) and the metal surface, which depicts chemisorption.

### 3.7 Effect of temperature

The influence of temperature on the corrosion inhibitive behavior of the two tested inhibitors were investigated by weight loss measurements in the temperature range 25–60 °C. Results obtained as depicted in Fig. 1 indicates that corrosion rate increased with increase in temperature in the absence and presence of the inhibitors. On the other hand, inhibition efficiency of the two inhibitors behave differently as temperature was increased. For BDMTI, IE increased with increase in temperature up to 50 °C and thereafter declined as the temperature was raised to 60 °C. With respect to HMBTI, IE is noted to increase with increasing temperature. Increase in inhibition efficiency with increase in temperature is often ascribed to chemisorption of the inhibitor molecules on the metal surface. The apparent decrease in IE as temperature was raised from 50 to 60 °C for BDMTI could be related to change of adsorption mode from chemisorption to physisorption caused by desorption of adsorbed inhibitor as a result of increased solution agitation due to higher rate of hydrogen gas evolution.^25^

**Fig. 8:**
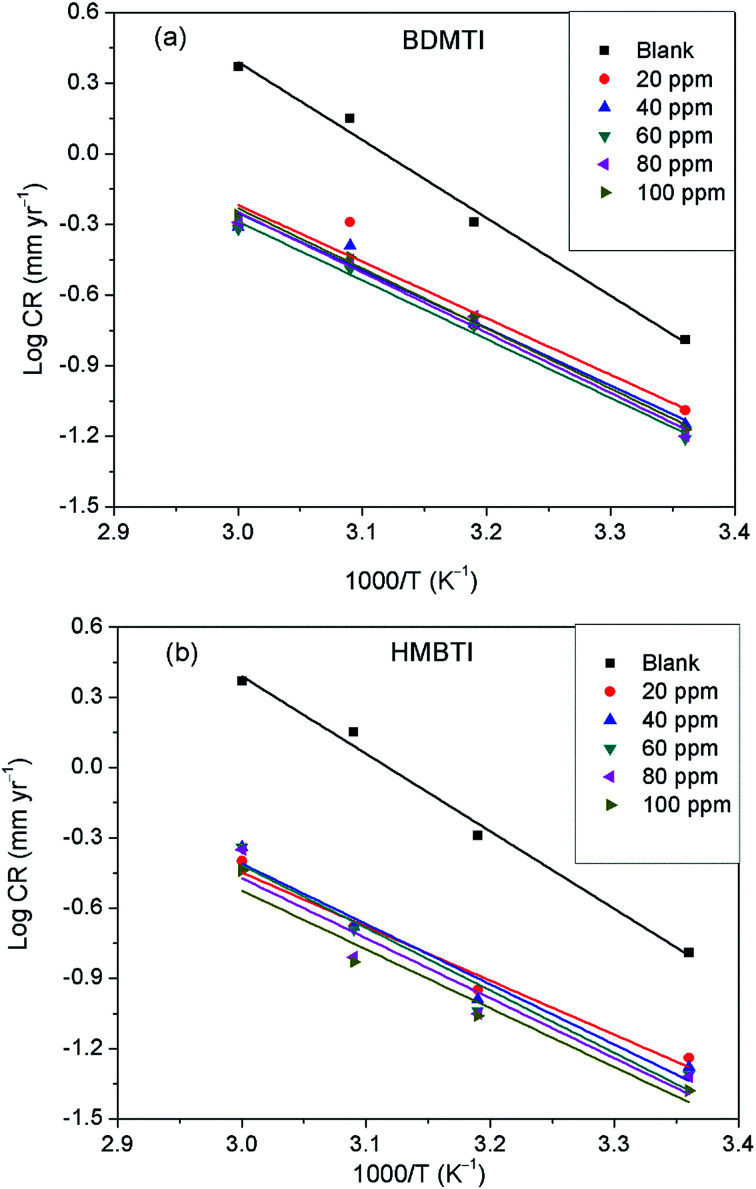
Arrhenius plot of log *C*_R_*versus* 1/*T* for API 5L X60 steel in 1 M HCl in the absence and presence of different concentrations of (a) BDMTI and (b) HMBTI.

**Fig. 9:**
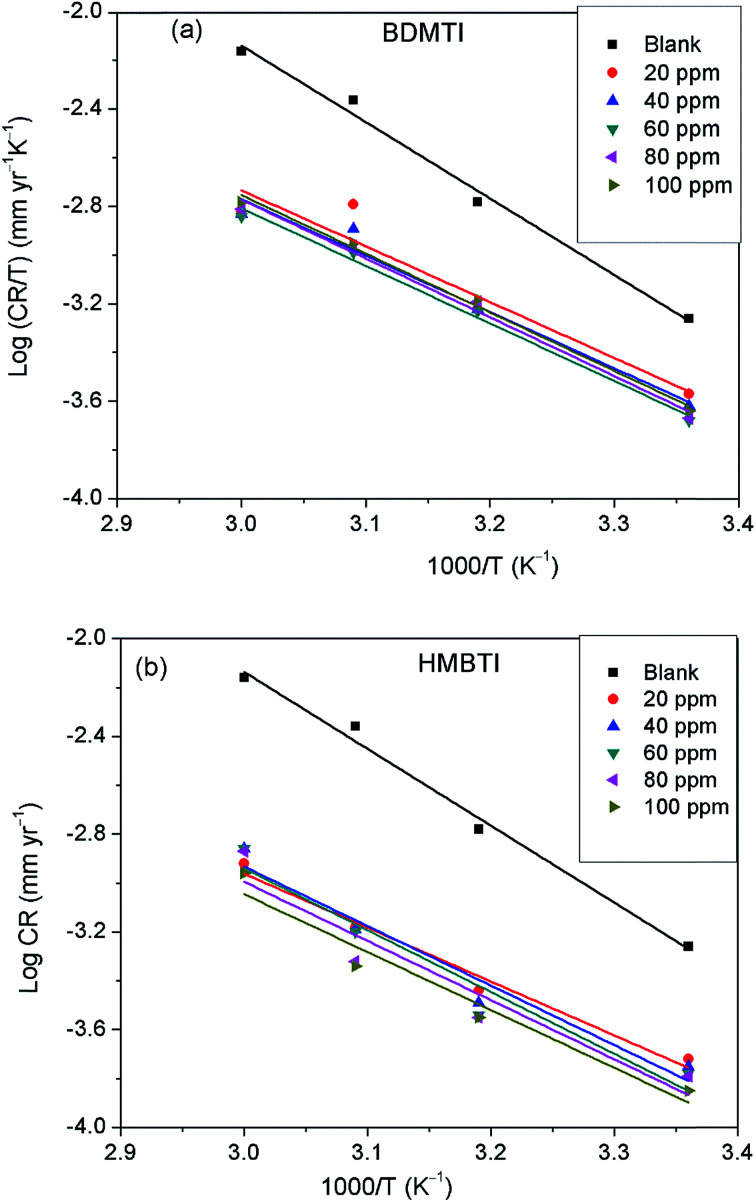
Transition state plot of log *C*_R_/*T versus* 1/*T* for API 5L X60 steel in 1 M HCl in the absence and presence of different concentrations of (a) BDMTI and (b) HMBTI.

The apparent activation energy (*E*_a_) for the corrosion process without and with BDMTI and HMBTI was computed from the Arrhenius equation:12
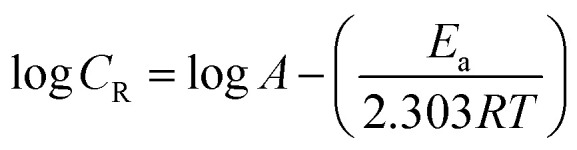
where *C*_R_ is the corrosion rate, *E*_a_ is the apparent activation energy, *R* is the molar gas constant, *T* is the absolute temperature, and *A* is the frequency factor. The Arrhenius plot of log *C*_R_ against reciprocal of absolute temperature (1/*T*) depicted in Fig. 8 gives a straight line with slope of −*E*_a_/2.303*R* from which the activation energy was computed and listed in Table 4. The enthalpy of activation (ΔH*) and the entropy of activation (ΔS*) for the corrosion of API 5L X60 in 1 M HCl were obtained from the transition state theory equation:13
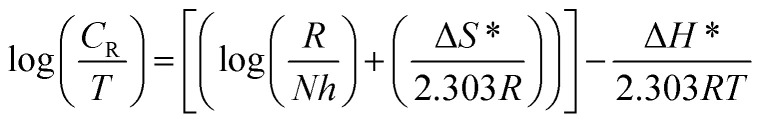
where *h* (6.626176 × 10^−34^ J s) is the Planck's constant, *N* (6.02252 × 10^23^ mol^−1^) is the Avogadro's number, *R* and *T* retain the earlier meanings. The plot of log(*C*_R_/*T*) against 1/*T* is shown to be linear in Fig. 9 from which ΔH* and ΔS* values were deduced from the slope and intercept of the plots respectively and also listed in Table 4.

**Table 4:** Activation parameters for API 5L X60 steel in 1 M HCl in the absence and presence of different concentrations of BDMTI and HMBTI

Inhibitor	Concentration (ppm)	*E*_a_ (kJ mol^−1^)	ΔH* (kJ mol^−1^)	−ΔS* (J mol^−1^ K^−1^)
	Blank	63.39	60.23	57.82
BDMTI	20	46.02	44.01	117.89
	40	46.85	44.38	117.47
	60	47.84	57.71	115.19
	80	49.02	45.37	111.54
	100	48.89	46.15	111.83
HMBTI	20	44.22	42.21	127.69
	40	49.26	46.61	113.93
	60	51.09	48.43	108.60
	80	49.02	46.37	115.85
	100	48.08	45.35	119.88

The data in Table 4 show that the values of *E*_a_ in the solution containing different concentrations of BDMTI and HMBTI are lower than that of the uninhibited solution. It has been reported^42–44^ that lower *E*_a_ value in the presence of inhibitors relative to the blank is ascribed to chemical adsorption while the reverse is interpreted to mean physisorption mechanism. The variation of *E*_a_ values in the inhibited solution with that of the uninhibited solution is consistent with the variation of inhibition efficiency with temperature. The activation entropy in all cases are negative, that is, in the absence and presence of the two tested inhibitors and it is seen to be more negative in the presence of BDMTI and HMBTI. In the literature, this has been interpreted to mean that the activated complex in the rate determining step represents an association rather than dissociation step, meaning that during the adsorption process, a decrease in the degree of disorderliness takes place on moving from reactants to the activated complex. Hence, orderliness is stepped up as reactants are transformed to activated complex.^45^ The enthalpy of activation values in all cases are positive, very close and exhibiting the same trend as *E*_a_. In the literature, the negative sign of ΔH* has been unequivocally associated with an exothermic adsorption process that can be either physisorption or chemisorption or combination of both,^46^ whereas the positive sign is linked to endothermic adsorption process which is attributed to chemisorptions. The positive sign of the enthalpy of activation as obtained in the present study reflects the endothermic nature of the steel dissolution process. This assumption is in agreement with the observed increase in inhibition efficiency with temperature indicating the chemisorption of the tested inhibitors on the metal surface.

### 3.8 Surfaces analysis

**Fig. 10:**
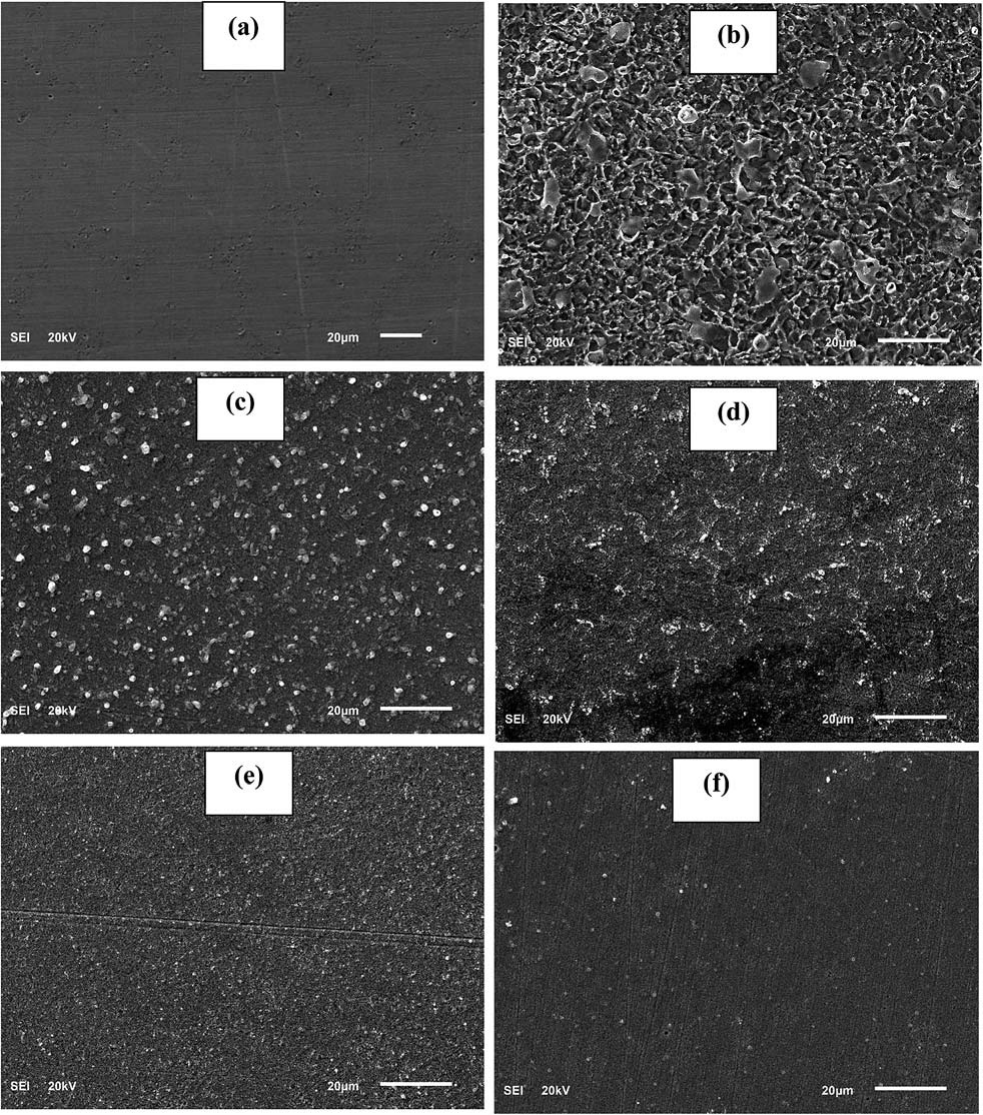
SEM images for API 5L X60 steel (a) in polished state (b) exposed to 1 M HCl solution (c) exposed to 1 M HCl solution containing 60 ppm DBMTI, (d) exposed to 1 M HCl solution containing 60 ppm HMBTI, (e) exposed to 1 M HCl solution containing 60 ppm DBMTI + 5 mM KI and (f) exposed to 1 M HCl solution containing 60 ppm HMBTI + KI at 25 °C for 24 h.

Fig. 10 depicts the alteration observed in polished API 5L X60 steel surface morphology after 24 h immersion in 1 M HCl in absence and presence of 60 ppm BDMTI, 60 ppm HMBTI and this concentration of the two tested inhibitors in combination of 5 mM KI. Surface micrographs of the steel specimens were obtained by SEM. As can be seen from Fig. 10b, the specimen treated with uninhibited 1 M HCl solution was highly corroded, and the surface became rough and porous. It is pertinent to concluded that the steel surface was highly damaged in absence of inhibitors due to fast and aggressive corrosion reaction. In the presence of both BDMTI and HMBTI (Fig. 10c and d), the API 5L X60 steel surface was much less damaged. A relatively smooth morphology of steel surface can be observed which shows the slowing down of the corrosion attack and the formation of a protective inhibitor film on the substrate surface. A smoother surface of API 5L X60 can be observed in Fig. 10e and f with BDMTI and HMBTI in combination with 5 mM KI respectively. This illustrates a less damage surface and can be linked to the co-adsorption of iodide ions and BDMTI and HMBTI molecules on the steel surface hence enhanced corrosion inhibition effect.

### 3.9 Quantum chemical calculations

**Fig. 11:**
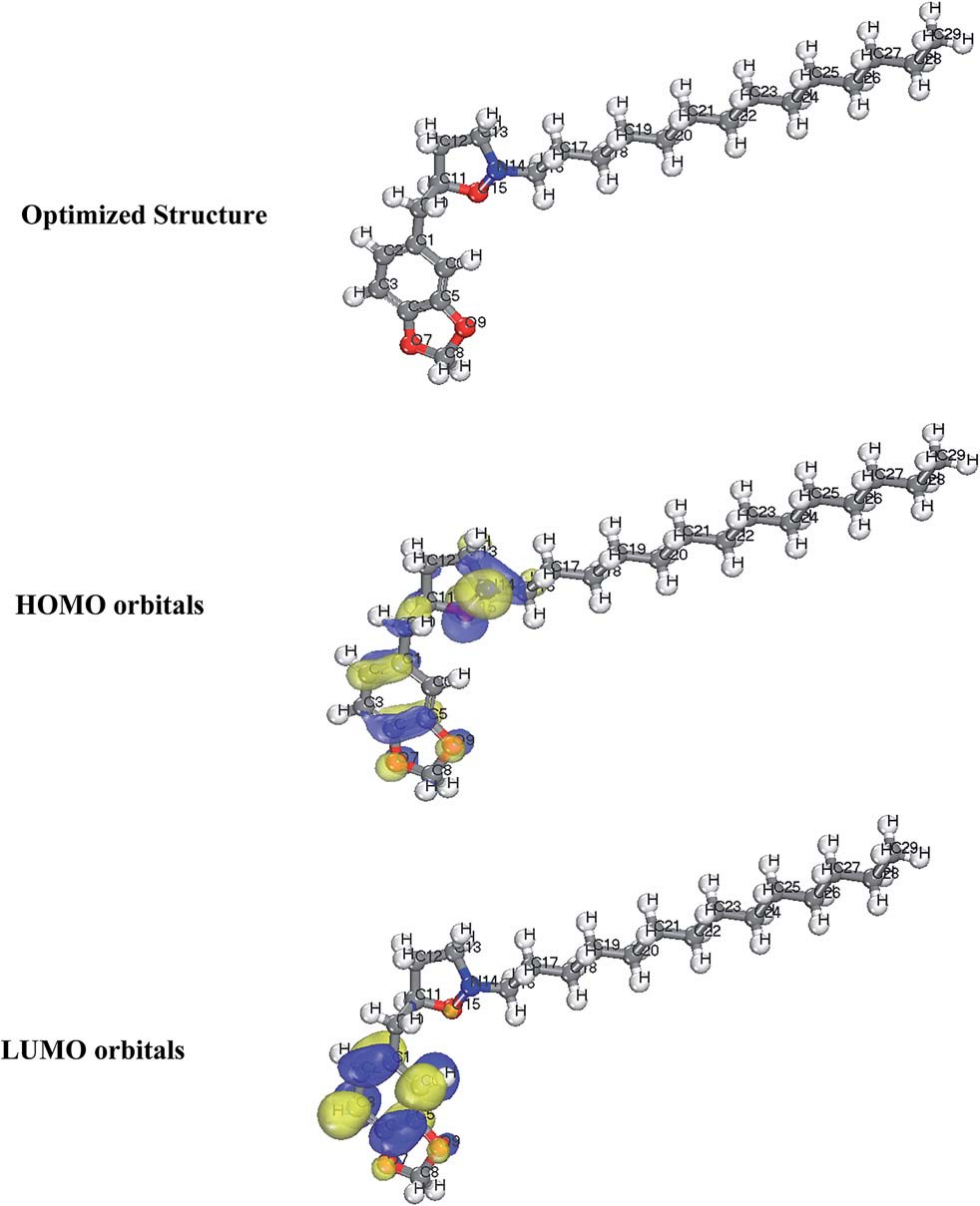
Optimized structure, HOMO and LUMO orbitals distribution of BDMTI.

**Fig. 12:**
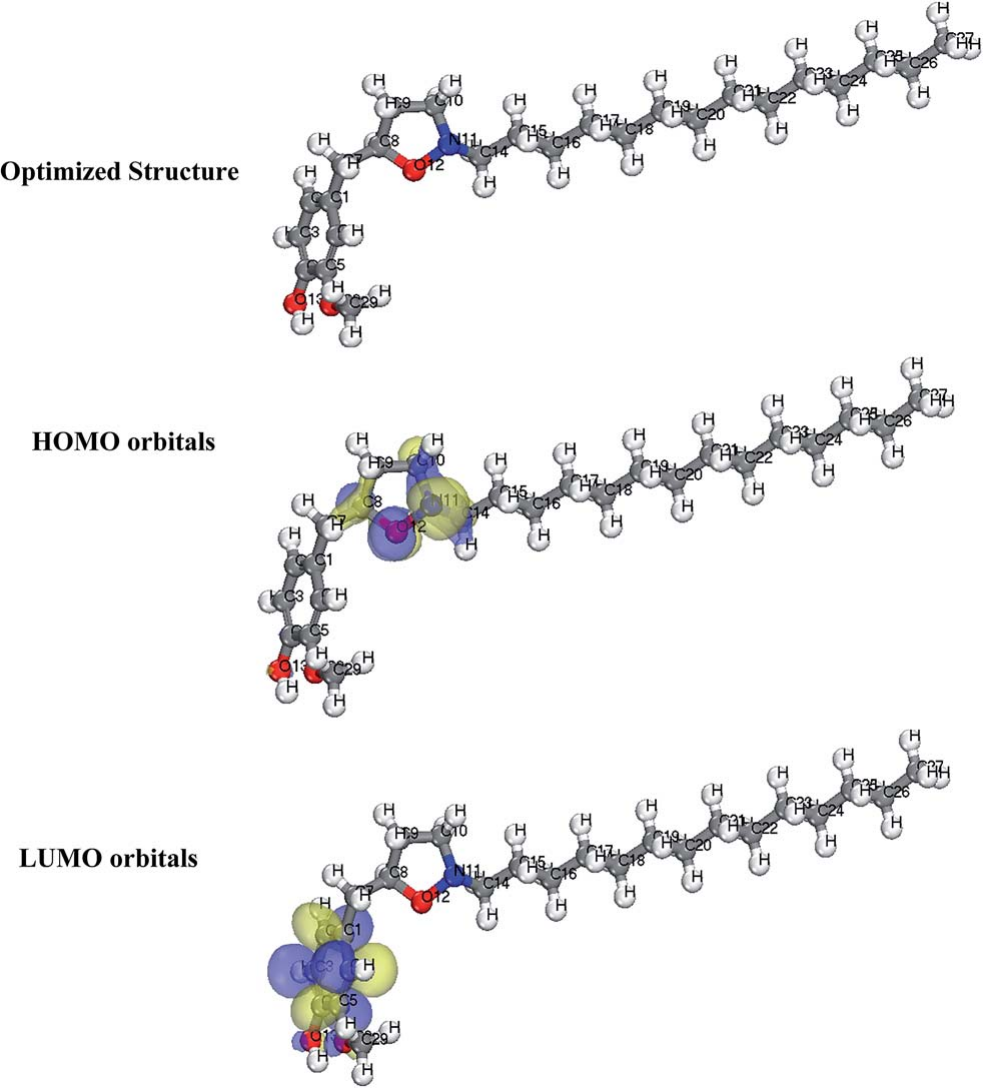
Optimized structure, HOMO and LUMO orbitals distribution of HMBTI.

Generally, HOMO and LUMO orbitals show the regions of molecule which can donate or accept electron, respectively. Molecule with large HOMO orbital is easy to donate electron to unoccupied d-orbital of a metal atom. Conversely, molecules with large LUMO orbital can easily accept electrons from a d-orbital metal atom. The optimized structure, HOMO and LUMO diagrams of BDMTI and HMBTI molecules using DFT model chemistry in aqueous phase are illustrated in Fig. 11 and 12, respectively. In the figures it is observed that the HOMO orbital for BDMTI are localized at 1,3-benzodioxole and isoxazolidine rings whereas the HOMO of HMBTI is only localize on the isoxazolidine ring. These are the regions donating electron to unoccupied d-orbital of metal. On the other hand, LUMO orbitals for BDMTI are found on 1,3-benzodioxole whereas that of HMBTI are localized on the 4-hydroxy-3-methoxybenzyl group. These regions can accept electron from metal surface. Thus, the HOMO and LUMO orbital analyses indicate that the isoxazolidine ring, benzodioxole ring, C_6_H_5_(OCH_3_) OH groups play an important role as active sites for the interaction of the two corrosion inhibitors with steel surface.

### 3.10 Monte Carlo simulations

**Fig. 13:**
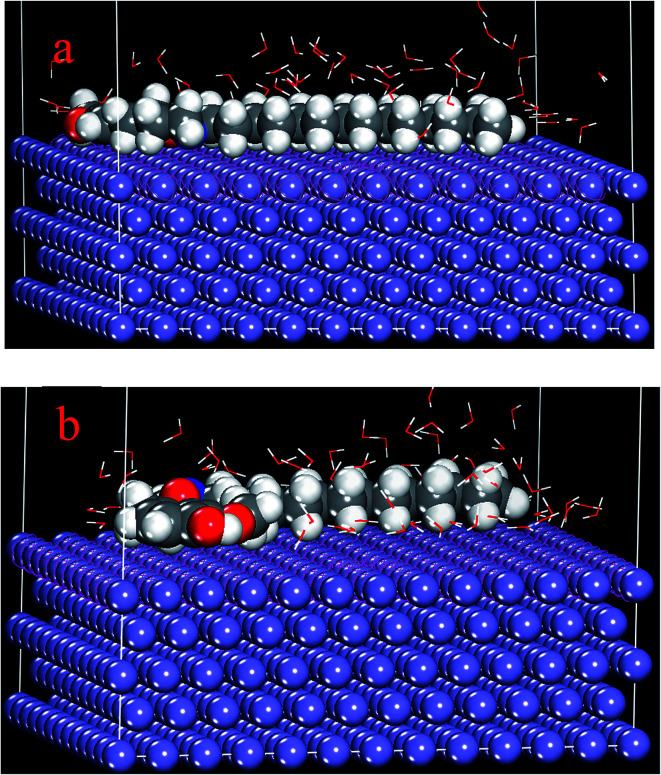
Stable equilibrium configuration of (a) BDMTI and (b) HMBTI adsorption on Fe (110)/100H_2_O interface using Monte Carlo simulation methodology.

Metropolis Monte Carlo simulations using simulated annealing procedure were further carried out to quantify the adsorption of BDMTI and HMBTI on steel surface. Fig. 13 shows (a) snapshot of the stable equilibrium configuration of BDMTI adsorption on Fe (110) surface and (b) snapshot of the stable equilibrium configuration of HMBTI adsorption on Fe (110) surface. The simulation was carried out in the presence of water (100H_2_O). Table 5 shows the output of the results obtained using Monte Carlo simulations. As can be seen in Fig. 13(a) and (b) both molecules are adsorbed in a parallel orientation to the metal surface in order to maximize contact. It has been reported that the higher the negative adsorption energies, the stronger the interaction of inhibitor molecules with metal surfaces.^47–49^ Results from Table 5, indicate that the order of adsorption energy is HMBTI > BDMTI. This shows that interaction of HMBTI with steel is expected to inhibit steel corrosion in acidic aqueous solution more than BDMTI. The theoretical results are in good agreement with the experiment.

**Table 5:** Parameters derived from Monte Carlo simulation of BDMTI and HMBTI adsorption on Fe (110)/100H_2_O interface (kcal mol^−1^)

Systems	Adsorption energy	Inhibition efficiency (%)
Fe (110)–BDMTI/100H_2_O	−266.26	61.32
Fe (110)–HMBTI/100H_2_O	−269.81	70.75

## 4 Conclusions

In the present investigation, two isoxazolidine derivatives namely 5-(benzo[*d*][1,3]dioxol-5-ylmethyl)-2-tetradecyl isoxazolidine (BDMTI) and 5-(4-hydroxy-3-methoxybenzyl)-2-tetradecyl isoxazolidine (HMBTI) were synthesized and characterized using FTIR, C-NMR, H-NMR, elemental analyzer. The corrosion inhibition effect of the synthesized compounds for API 5L X60 steel in 1 M HCl solution was evaluated using weight loss and electrochemical techniques complemented with surface analysis of the corroded steel samples immersed in uninhibited and inhibited solutions with SEM. Theoretical studies was also undertaken to evaluate the adsorption/binding of the inhibitor molecules onto the steel surface. From the results obtained, the following conclusions could be drawn:

(1) Two new isoxazolidines having hydrophobic alkyl chain and π-electron-rich aromatic rings of naturally occurring safrole and eugenol have been synthesized using single-step nitrone cycloaddition reactions.

(2) The two synthesized compounds act as corrosion inhibitor for API 5L X60 in 1 M HCl but with HMBTI showing superior performance.

(3) Corrosion inhibition performance is found to depend on the concentration of the inhibitors and temperature.

(4) Addition of small amount of iodide ions improves the inhibition efficiency of the two inhibitors due to co-adsorption of the iodide ions and the inhibitors on the steel surface.

(5) The co-adsorption of the inhibitors and iodide ions was found to be competitive in nature as confirmed from synergistic parameter (*S*_1_) which was less than unity especially at higher temperature.

(6) Results of potentiodynamic polarization studies revealed that the two inhibitors functions as a mixed type corrosion inhibitor.

(7) Corrosion inhibition occurs by virtue of adsorption of the inhibitor molecules on the steel surface which was found to accord with Langmuir adsorption isotherm model.

(8) Results from the experimental and theoretical considerations are in good agreement confirming that HMBTI is a better corrosion inhibitor than BDMTI.

## Conflicts of interest

There are no conflicts to declare.

## Supplementary Material
